# The impact of urban rain on the changes of bare and artificially patinated bronze during 9-year exposure

**DOI:** 10.1007/s11356-024-33369-9

**Published:** 2024-04-19

**Authors:** Tadeja Kosec, Mirjam Bajt Leban, Polonca Ropret, Matjaž Finšgar

**Affiliations:** 1 Slovenian National Building and Civil Engineering Institute, Dimičeva ulica 12, 1000 Ljubljana, Slovenia; 2 Research Institute, Institute for the Protection of the Cultural Heritage of Slovenia, Poljanska cesta 40, 1000 Ljubljana, Slovenia; 3 Faculty of Chemistry and Chemical Engineering, University of Ljubljana, Večna Pot 113, 1000 Ljubljana, Slovenia; 4 Faculty of Chemistry and Chemical Engineering, University of Maribor, Smetanova ulica 17, 2000 Maribor, Slovenia

**Keywords:** Bronze, Urban environment, Outdoor exposure, Patina, Corrosion, Patina characterisation

## Abstract

Atmospheric pollutants in the air form acid rain which interacts with bronze surfaces exposed in urban outdoor environment. In this study, different types of patinas on bronze were investigated during and after 9 years of exposure to urban environment in moderately polluted continental city. Natural bronze patina and artificial brown sulphide, green chloride, and green–blue nitrate patinas were investigated. Visual assessment was carried out at defined periods. After 9 years of exposure, an electrochemical study was performed to investigate the electrochemical activity of the patinas in artificial urban rain. Additionally, the patinas were characterised using a variety of techniques, including metallographic examination, scanning electron microscopy/energy dispersive X-ray spectroscopy, Raman spectroscopy, X-ray diffraction analysis, X-ray-photoelectron spectroscopy, and time-of-flight secondary ion mass spectrometry to analyse the surface morphology, chemical composition, and stratigraphic features of the patinas. Evolution of the patinas was shown to be a result of both, the composition of the acid rain and the hydrophobicity of the patinated surfaces.

**Supplementary Information:**

The online version contains supplementary material available at 10.1007/s11356-024-33369-9.

## Introduction

Under exposure of unprotected bronze to atmospheric conditions, a bronze patina forms, which is a smooth homogenous layer across the surface of bronze (Scott 2002). The terms patina and corrosion differ in that, when the aesthetics are preserved, the evolution of a patina is expected (i.e. this change is desired), whereas sometimes rain can cause leaching at the surface, leading to the severe dissolution or accumulation of a patina. When the patina is not formed homogeneously, and does not preserve the texture or appearance intended by the artist or sculptor, corrosion occurs, and restoration is required.

Numerous studies on bronze patinas developed in urban environment conditions have been published previously (FitzGerald et al. 2006; Chiavari et al. 2007; Robbiola et al. 2008; Bernardi et al. 2009; Dillmann and European Federation of Corrosion 2013; Chelaru et al. 2014; Masi et al. 2017). A natural pale green patina stands for the enrichment of tin compounds in pale green patina, which has been attributed to the selective dissolution of copper and zinc in bronze exposed outdoors (Chiavari et al. 2007; Robbiola et al. 2008), and is significantly affected by the pH of the environment (Robbiola et al. 2008). The patinas developed on bronze exposed to an outdoor environment depend greatly on the type of material and the aggressiveness of the atmosphere to which it is exposed (Scott 2002). It was reported previously that patinas on copper roofs exposed to an urban environment developed a layer of cuprite (Cu_2_O) up to 6 µm thick, covered with a layer of brochantite [Cu_4_(SO_4_)(OH)_6_] up to 15 µm thick, with the thickness varying according to the number of years of exposure (FitzGerald et al. 2006). Comparing copper and Cu_4_Sn, it was found that, in general, much thinner natural patinas developed on Cu_4_Sn than on copper (2 µm vs. up to 10 µm) in urban environments, whereas in marine environments, the patinas on Cu_4_Sn were thicker (20–40 µm) than those on copper (15–20 µm) (Chang et al. 2019).

The thickness of patinas on bronze can be determined using a variety of methods, including metallographically, by preparing cross-sections (Robbiola et al. 1998; Chang et al. 2020; Manti and Watkinson 2022), through FIB milling (Masi et al. 2019; Kosec et al. 2022), electrochemically, using coulometric measurements (Souissi et al. 2007), and through the measurement of Eddy current (Letardi et al. 2016). Only few publications examining the cross-section of bronze exposed to an outdoor environment exist, due to the fact that bronze statues cannot be sampled invasively. Moreover, outdoor studies of samples over very long periods of exposure are rare, due to the costs connected with long-term studies and the scientific ‘urge’ to push and accelerate investigations. A group of researchers from Bologna University have therefore developed an ageing method whereby exposure to an outdoor environment is simulated through dropping and wet and dry tests (Bernardi et al. 2009).

Artificial patinas are also called chemical, aesthetic, artificial, artists’, or intentional patinas. Artificial patinas are applied to bronze using chemical solutions and/or heat, which react with the surface to form a thin layer of coloured corrosion products with varying effects (Hughes and Rowe 1991; Morgan 1999). These patinas, however, also change upon exposure to different environments. A systematic study of patination techniques on bronze is therefore required (De Oliveira et al. 2009; Rahmouni et al. 2009; Otmačić Ćurković et al. 2012). Multiple studies have been conducted to characterise artificial patinas electrochemically (De Oliveira et al. 2009; Rahmouni et al. 2009; Hernández et al. 2011; Otmačić Ćurković et al. 2012). Ageing has also been examined through the application of various techniques (Kosec et al. 2010a, 2012; Ropret and Kosec 2012; Qafsaoui et al. 2018; Kapitanović and Ćurković 2022), such as exposure to conditions in an environmental chamber (Ropret and Kosec 2012; Kapitanović and Ćurković 2022) and immersion in a solution (Kosec et al. 2010a, 2012; Qafsaoui et al. 2018). Numerous studies investigating brown patinas exist (Kosec et al. 2010a; Masi et al. 2019; Kapitanović and Ćurković 2022), but far fewer studies have examined other types of artificial patina. Sulphide patinas on different bronzes have also been investigated following ageing in various environments, using both electrochemical and spectroscopic techniques (Kapitanović and Ćurković 2022).

It is difficult to ascertain the exact number of bronze statues exposed to an outdoor environment in Slovenia. The Ministry of Culture holds a central register, the ‘Registry of Immovable Cultural Heritage’, which identifies the details of any given bronze statue using its identity number. There were 219 bronze items identified within the Municipality of Ljubljana, including 15 bronze statues of national or local importance, 34 bronze busts, 13 fountains, 40 versatile commemorative plaques, several bronze models of the city, and many World War 2 memorial statues.

To date, there are no records in place detailing restoration works conducted on important bronze statues. As a result, in 2008, the Municipality of Ljubljana (Applied project ‘The protection of bronze statues in Ljubljana’) ordered an investigation into the protection of outdoor bronze statues in the Ljubljana area. In order to develop knowledge regarding the properties of patinas and methods for their protection, various short- and long-term studies were conducted within this project over the following several years. The patinas studied with respect to the future restoration of bronze statues with various patinas were as follows: a brown (sulphide) patina, commonly used for new works, a green-patinated brown patina, used to resemble an antique appearance, and an alternative green patina—blue-green nitrate patina. The stability and long-term properties, as well as the ease of patination, were investigated by exposing the prepared samples to outdoor conditions for extended periods of time.

This paper aims to investigate the impact of a changing urban atmosphere resulting from an increasing amount of traffic and particulate matter (PM), on patinated bronze surfaces. An interesting feature of the environment in Ljubljana is a relatively high concentration of chloride ions, which results from the use of deicing salts in its cold winters and a 3-month period of heavy fog during the winter months. This affects pollution in particular and, in addition to the changing concentrations of sulphates and nitrates, further affects bronze surfaces and patinas.

The present study deals with the evolution and degradation of chemically prepared patinas to bronze exposed to a polluted urban environment for a period of 9 years. The evolution of the chemically prepared patinas was compared to that of a patina developed naturally in the same environment. Both the surface and cross-sections of the 9-year-old bronze patinas were investigated in this study. Different spectroscopic techniques were used to investigate the structure, morphology, and degradation of the natural and artificial bronze patinas. The aim of this work was to determine the thickness of the patinas using metallographic examination and to characterise the composition of the patinas after 9 years of exposure using Raman spectroscopy, XRD, and XPS techniques. The chemical composition, surface morphology, and cross-sections of the patinas were further studied using scanning electron microscopy/energy dispersive X-ray spectroscopy (SEM/EDXS). Electrochemical methods were used to analyse the susceptibility of the 9-year-old patinas to corrosion.

## Materials and methods

### Sample preparation and exposure to the urban environment

The bronze samples were sand cast in moulds with dimensions of 100 mm × 100 mm × 5 mm. The composition of the alloy investigated is similar to that of quaternary bronze (CuSnZnPb), which was used for casting bronze statues at the beginning of the twentieth century. The composition of the bronze was analysed using arch optical emission spectroscopy (SPECTRO MAXx, Spectro Analytical Instruments GmbH, Kleve, Germany), with the elemental composition being 5.27 wt.% Sn, 3.77 wt.% Zn, 0.08 wt.% Pb, and 90.70 wt.% Cu. The surfaces of the bronze plates were blast-cleaned by sand to remove any mill scale or foreign matter then cleaned using dry compressed air.

Three types of artificial patina were prepared. A brown sulphide patina was obtained on the bronze by brushing the hot surface with a 3 wt.% K_2_S solution (Hughes and Rowe 1991). The surface was then rinsed with tap water in order to represent real application procedure. A green chloride patina was applied over the warm surface (50 °C) of a brown patina by brushing it with a freshly prepared solution of 30 g (NH_4_)_2_CO_3_ and 30 g of NH_4_Cl mixed in 100 mL of deionised water. Using this procedure, a green chloride patina developed under conditions of high humidity. The nitrate patina was obtained by brushing the hot brown sulphide patina with a 3 wt.% Cu(NO_3_)_2_ solution. A homogenous blue-to-green colour was developed by brushing the surface multiple times. The amount of solution applied was controlled by the number of strokes made with a wet brush, and the temperature was also controlled.

### Conditions of exposure

The samples were exposed to natural ageing on a rooftop of the author’s institution (Slovenian National Building and Civil Engineering Institute) in Ljubljana, the capital city of Slovenia, for 9 years. Specifically, coupons of bronze were mounted on a rack inclined 75° from the horizontal, facing south.

The atmosphere in Ljubljana is a typical urban environment, with relatively cold winters, heavy fog during the winter, and moderate rainfall throughout the rest of the year.

According to data from the Slovenian Environment Agency, the average composition of rain in the Ljubljana region over the period 2012 and 2021 is given in Table 1.

**Table 1:** Environmental data (yearly averages) for Ljubljana between 2012 and 2021

Year	T (°C)	Precipitation (mm)	PM 10 (µg/m^3^)	Contaminants in rain		
				SO_4_^2−^ (mg/L)	NO_3_^−^ (mg/L)	Cl^−^ (mg/L)
2012	12.0	1339	28.4	1.46	2.41	0.595
2013	11.6	1531	26.9	1.62	2.11	0.415
2014	12.6	1841	23.2	/	/	/
2015	12.2	1106	26.3	/	/	/
2016	11.8	1317	25.6	0.95	1.80	0.384
2017	11.9	1531	25.8	0.83	1.88	0.466
2018	12.5	1377	25.0	1.14	1.88	0.509
2019	12.5	1379	23.6	1.12	1.78	0.434
2020	12.1	1262	20.8	0.92	1.82	0.664
2021	11.5	1442	21.0	0.86	1.64	0.604

### Electrochemical investigation

A simulated urban rain solution was prepared for electrochemical investigation, with a concentration 1000-times higher than the average concentration of rainwater. It contained 685.7 mg/L SO_4_^2−^, 287.0 mg/L Cl^−^, and 943.2 mg/L NO_3_^−^, and had a pH of 5.4 and conductivity of 3.716 mS/cm.

A three-electrode corrosion cell was constructed in-lab by glueing a glass tube onto a bronze plate. A carbon electrode served as the counter electrode, and an Ag/AgCl (3 M KCl) electrode as the reference. Contact with the bronze plate was established, and thus the working electrode was connected. The volume of the solution in the glass tube was 19.5 mL (the setup is presented in Figure S1 in the Supplementary Information).

Firstly, open circuit potential was measured, for a minimum of 2 h and until a steady state was achieved. Polarisation resistance ± 10 mV vs. corrosion potential (*E*_corr_) was then measured, using a scan rate of 0.1 mV/s. The results were analysed using Echem Analyst software, and electrochemical tests run on a Gamry Ref 600 + potentiostat/galvanostat (Gamry Instruments Inc, Warminster, USA). At least three measurements were conducted for each sample.

### Surface characterisation techniques

#### XRPD analysis

The surface composition of the patinas on the bronze specimens was analysed using an X-ray diffractometer (Empyrean, PANalytical, Netherlands) with Cu K_α_ radiation. X-ray powder diffraction (XRPD) measurements were performed at room temperature with a Cu tube tension of 45 kV and a tube current of 40 mA, using a 2θ step of 0.013° and a measurement time of 150 s per step. Data was collected over a 2θ range of 10 to 70°. The results were analysed using HighScore Plus (PANalytical, Netherlands) diffraction software, using the Powder Diffraction File PDF-4 + (2020, ICDD, USA) database as a source of reference data.

#### Raman spectroscopy analysis

Raman spectroscopy analysis of the natural bronze patina and the brown sulphide, green chloride, and green–blue nitrate patinas developed following 9 years of urban environment exposure was performed with a Horiba Jobin Yvone LabRAM HR800 Raman spectrometer coupled to an Olympus BXFM optical microscope using 514 nm laser excitation lines. The spectra were recorded using a × 100 objective lens and a 600 grooves/mm grating, which gave the spectral resolution of ca. 2 cm^−1^ pixel^−1^. The power at the samples was set to 0.14 mW using neutral density filters. A multi-channel, air-cooled CCD detector was used, with integration times between 5 and 20 s, and the spectral range was set between 50 and 4000 cm^−1^. Wave number calibration was performed using a silicon wafer.

#### Metallographic analysis, SEM/EDXS, and contact angle measurements

Metallographic sections were prepared by cold cut cross-sections of the specimens, hot-mounted in epoxy resin and grinded using 320-, 600-, 1000-, 2400-, and 4000-grit SiC paper; a final polishing with 0.05 µm was made by colloidal silica. Observations were conducted using a Zeiss Axio Imager Z2m microscope with Zen software (Carl Zeiss AG, Aalen, Germany), using both bright field and polarised light.

A JEOL JSM-IT500 electron microscope equipped with an Aztec Live Advanced ULTIM 65 EDXS detector (Oxford Instruments, Abingdon, UK) was used to observe the patinas on the metallographically prepared cross-sections and the morphology of the surfaces, using an accelerating voltage of 20 keV. SEM/EDXS analysis was performed on the surface of each of the specimens following exposure to the urban environment for 9 years.

Contact angles were measured using an FTA 1000 DropShape Instrument B FrameSystem (First Ten Angstroms, Newark, USA). The static contact angle was measured on a 2 µL droplet of distilled water placed on the surface of each patina, with at least three different measurements performed per patina. Contact angle measurements were made at the end of the exposure period.

#### ToF–SIMS measurements

ToF–SIMS measurements were performed with an M6 device (Iontof, Munster, Germany). The primary ion beam was 30 keV Bi^+^ with a target current of 1.2 pA. 2D imaging was performed on 1.0 by 1.0 mm. Calibration of the spectra was performed using the peaks at known mass-to-charge *m*/*z*.

#### XPS measurements

XPS measurements were performed using a Supra + instrument (Kratos, Manchester, UK) equipped with an Al K_α_ source, a monochromator, and a charge neutraliser. The take-off angle of the analysis was 90°. The charge neutraliser was turned on during the acquisition of the XPS spectra. The binding energy scale was corrected using the C–C/C-H peak in the C 1 s spectrum at 284.8 eV. Data were acquired and processed using ESCApe 1.4 software (Kratos, Manchester, UK). The pass energy used to acquire high-resolution (HR) and survey spectra was 40 eV and 160 eV, respectively. Quantification was performed by normalising the surface atomic concentrations to 100.0 at.% after carrying out a Shirley background subtraction (Shirley 1972).

## Results

### Surface observation and contact angle measurements

**Fig. 1:**
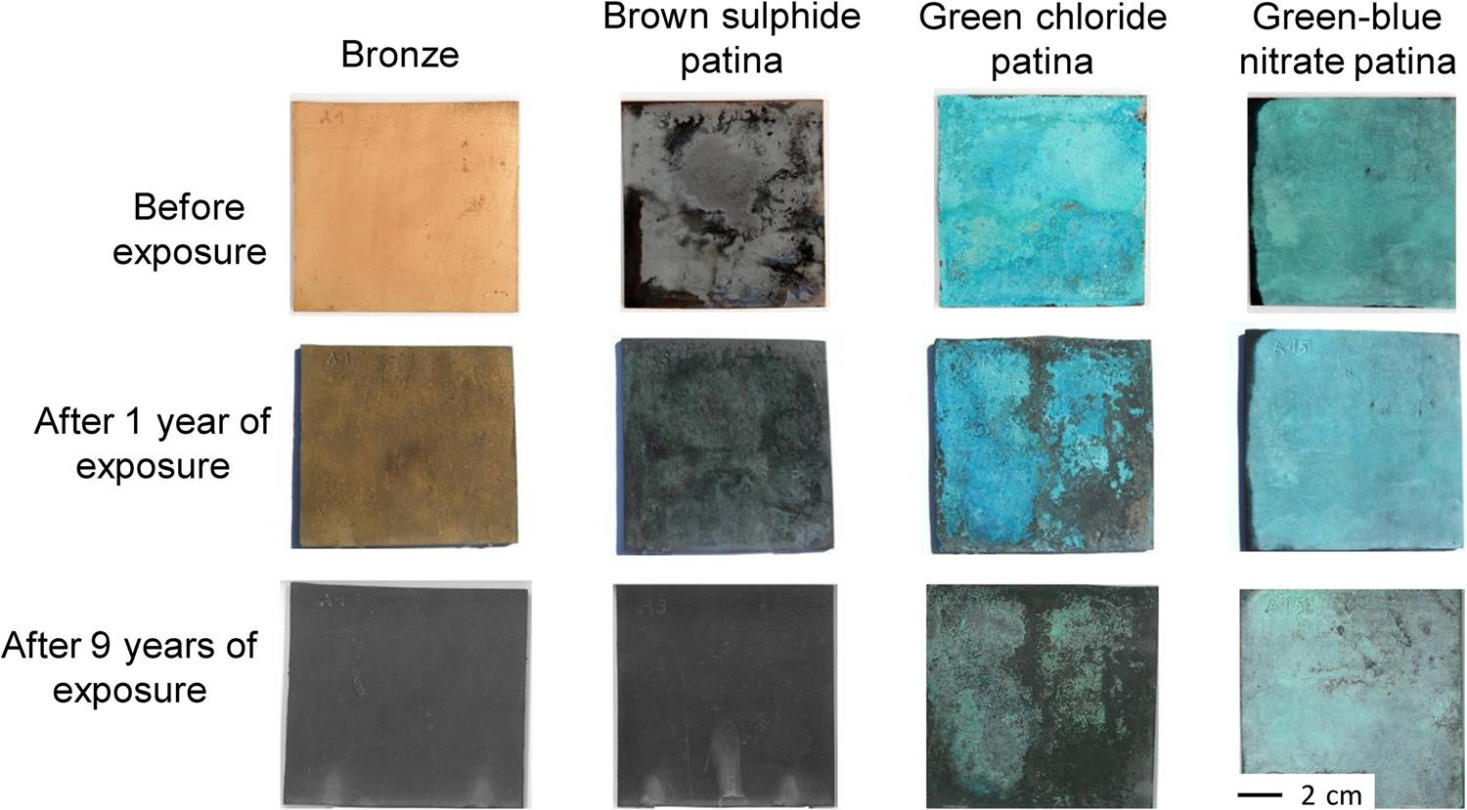
Photographic images of the bronze and the brown sulphide, green chloride, and green–blue nitrate patinas before and after exposure to an urban environment for either 1 or 9 years

The non-patinated (bare) bronze and chemically prepared patinated bronzes were exposed to an urban environment for a period of 1 and 9 years. Photographs of the bronze surfaces are presented in Fig. 1, comparing the freshly prepared patinas with images of the patinas after 1 and 9 years of exposure. After 1 year of exposure, the surface of the natural bronze patina was slightly darker. The brown sulphide patina had not changed significantly after 1 year of exposure, with the only change in appearance being the loss of its shiny texture. As the green chloride patina was initially thick and flaky, after 1 year it had spalled off in some areas, especially at the edges and in the centre of the plate, uncovering the underlying sulphide patina. The green–blue nitrate patina was homogeneous and became pale blue after 1 year of exposure to the urban environment.

After 9 years of exposure, the bronze surface darkened, forming a homogenous dark brown surface. The brown sulphide patina remained brown. In the green chloride patina, the extent of the green surface reduced even further, while the green–blue nitrate patina became visually thinner over time.

The contact angle of the natural bronze patina, measured at different points of the surface, was 89.6 ± 7.8° (average value ± standard deviation). On the brown sulphide patina, the contact angle was higher (138 ± 2.5°). The green chloride patina had a contact angle of 79.5 ± 4.4°, while the green–blue nitrate patina had the lowest contact angle measured, at 51.5 ± 3.2°.

All the samples were then extensively investigated to study the electrochemical activity, thickness, and composition of the patinas.

### Electrochemical investigation

**Fig. 2:**
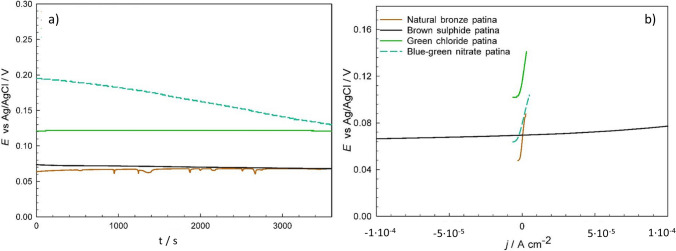
**a** Open circuit potential measurements vs. time (*t*) and **b** linear polarisation measurements for the 9-year-old patinas in 1000-times concentrated urban rain (pH 5.4, conductivity 3.716 mS/cm)

The electrochemical activity of the 9-year-old patinas was studied in order to define their electrochemical stability in urban environments. Electrochemical tests were conducted in a concentrated simulated acid rain solution. A photograph of the electrochemical setup is given in the Supplementary Information (Figure S1). Open circuit potential and linear polarisation measurements are presented in Fig. 2, while the electrochemical parameters deduced are given in Table 2.

**Table 2:** Electrochemical parameters for the various patinated bronzes after 9 years of exposure to the concentrated simulated urban rain solution

Sample	*E*_OCP_/V	*R*_p_/(kΩ cm^2^)
Natural bronze patina	0.067	12.50
Brown sulphide patina	0.068	0.05
Green chloride patina	0.121	6.96
Green–blue nitrate patina	0.130	5.02

Figure 2 b shows that polarisation resistance was highest for the natural bronze patina, with an *R*_p_ of 12.50 kΩ cm^2^, meaning that this patina was more resistant against general (uniform) corrosion than the other patinas. The brown sulphide patina had a lower *R*_p_ value, meaning it is less resistant to corrosion. The *R*_p_ for the green chloride patina was 6.96 kΩ cm^2^; its OCP value was stable during the entire exposure to concentrated simulated urban rain solution and at more positive potential than that for the sulphide and natural bronze patinas, with a value of 0.121 V after 3600 s of immersion. The green–blue nitrate patina had the most positive OCP value (0.130 V after 3600 s of immersion), and a *R*_p_ value of 5.02 kΩ cm^2^. These results show that the surfaces of the various patinas differ in terms of their electrochemical characteristics and corrosion resistance.

### Metallographic and SEM/EDXS observation of the sample cross-sections

**Fig. 3:**
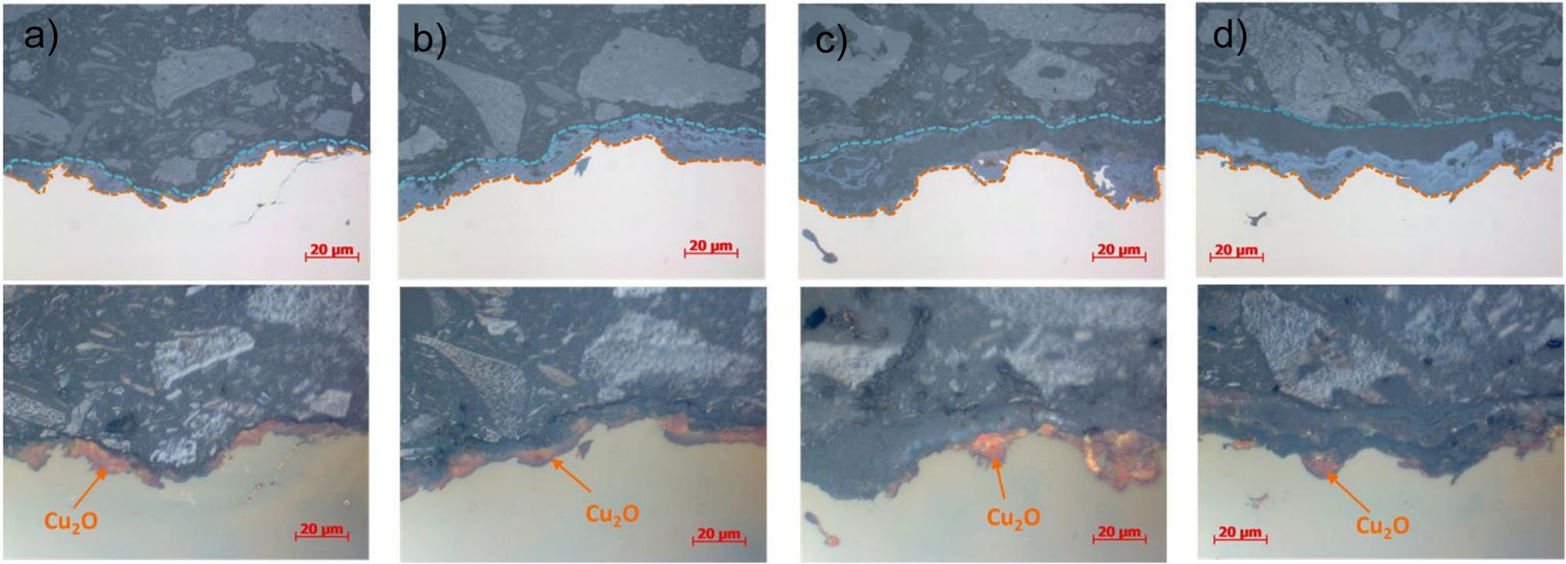
Optical micrographs of metallographic cross-sections of the bronze patinas developed over 9 years of exposure to urban environment: **a** natural bronze patina, **b** brown sulphide patina, **c** green chloride patina, and **d** green–blue nitrate patina. Upper images: bright field; lower images: polarised light

Figure 3a shows the cross-section of a natural bronze patina placed in an urban environment for 9 years. This patina primarily consisted of cuprite (Cu_2_O), which was observed as a pale red to orange layer under polarised light. The natural bronze patina layer is present throughout the cross-section, but the thickness of the layer varies from 5 to 10 µm (Fig. 3a).

Figure 3b shows the brown sulphide patina layer that developed on the sulphide-patinated bronze over the course of 9-year exposure to the urban environment. It consists of a layer up to 17 µm thick, in which the inner layer consists of Cu_2_O islands of up to 10 µm thick.

Compared to the brown sulphide patina, the green chloride patina following 9 years of exposure is thicker (20–45 µm; Fig. 3c), with Cu_2_O islands up to 20 µm thick present at the interface between the bronze and the patina.

The structure of the green–blue nitrate patina after 9 years is especially interesting due to its two-layer structure with the presence of small cuprite islands. The green–blue nitrate patina was up to 30 µm thick, but contained a lower amount of cuprite compared to the brown sulphide and green chloride patinas (Fig. 3d) as observed from lower positioned images (see orange arrows).

It is important to note that the presence of Cu_2_O observed in the cross-sections of the natural bronze patina is due to exposure to the urban environment, while in the case of the chemical patinas, Cu_2_O is present due to the patination procedure. This was confirmed in our previous studies (Masi et al. 2019; Kosec et al. 2022).

**Fig. 4:**
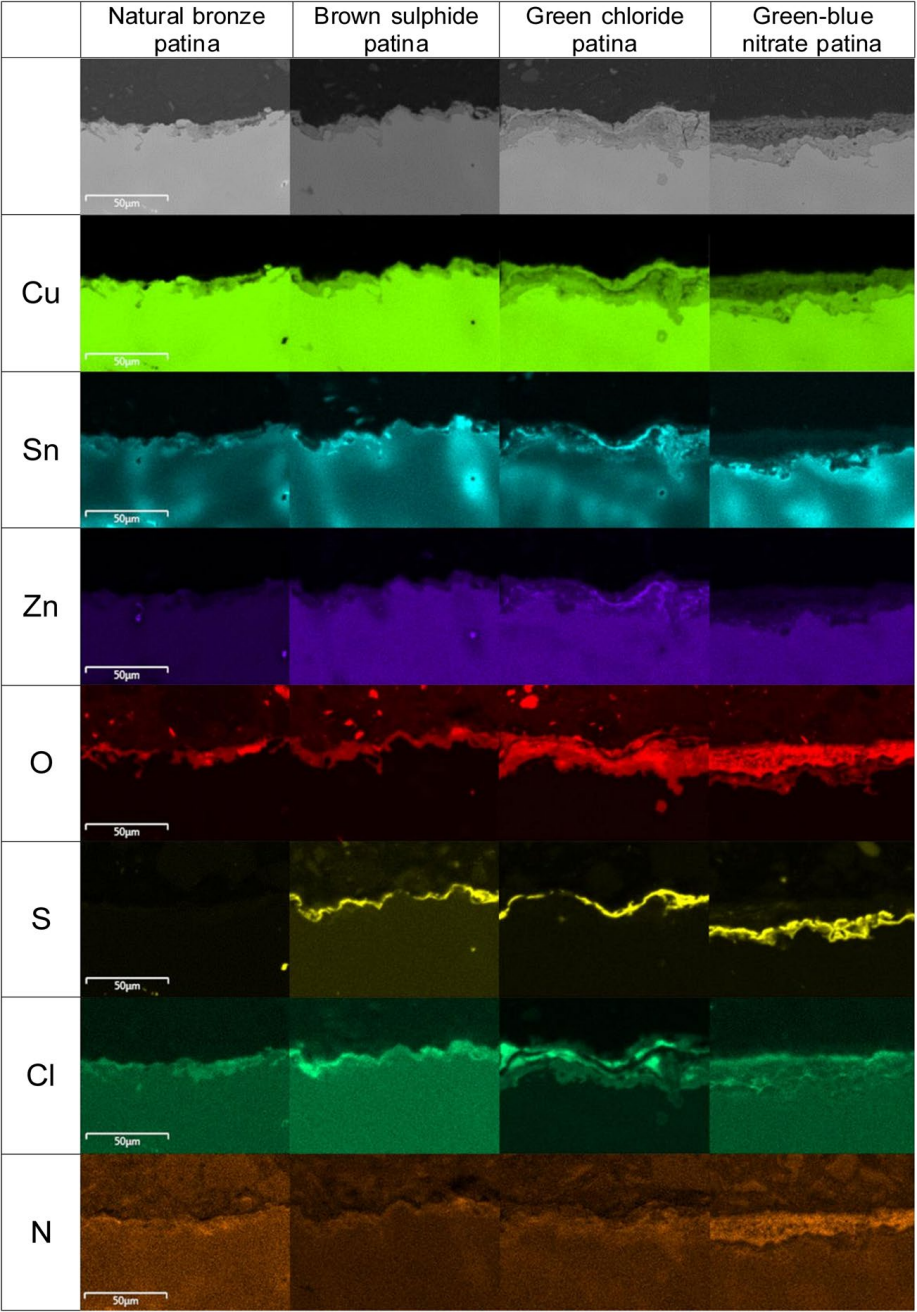
Cross-section SEM images (top) and corresponding EDXS mapping analysis for Cu, Sn, Zn, O, S, Cl, and N for the natural bronze, brown sulphide, green chloride, and green–blue nitrate patinas after 9-year exposure to an urban environment

Figure 4 shows SEM images of the areas analysed and EDXS elemental mapping distributions for Cu, Sn, Zn, O, S, Cl, and N in both the natural bronze patina and the chemical patinas following 9 years of urban environmental exposure. Cu and O were detected in abundance in the natural bronze patina, and they are present throughout the entire area analysed.

In the case of the brown sulphide patina, an interlayer containing sulphur exists between the bronze and the patina. Oxygen was observed both within and on top of the sulphide layer (where the S signal is of high intensity). EDXS mapping for O and Sn shows a thick and porous green chloride patina. S was also found at the uppermost position of the green chloride patina, which is additionally enriched with chlorine. The signal for O is intense throughout the entire chloride patina layer. The green–blue nitrate patina consists of two layers, with sulphur being the primary constituent of the first layer. The upper layer of the patina is also enriched with N, and it showed a more intense signal for O than was seen in the lower layer. Observation reveals a distinct zone in the form of a sulphide layer, which contains higher amounts of sulphur and copper.

**Fig. 5:**
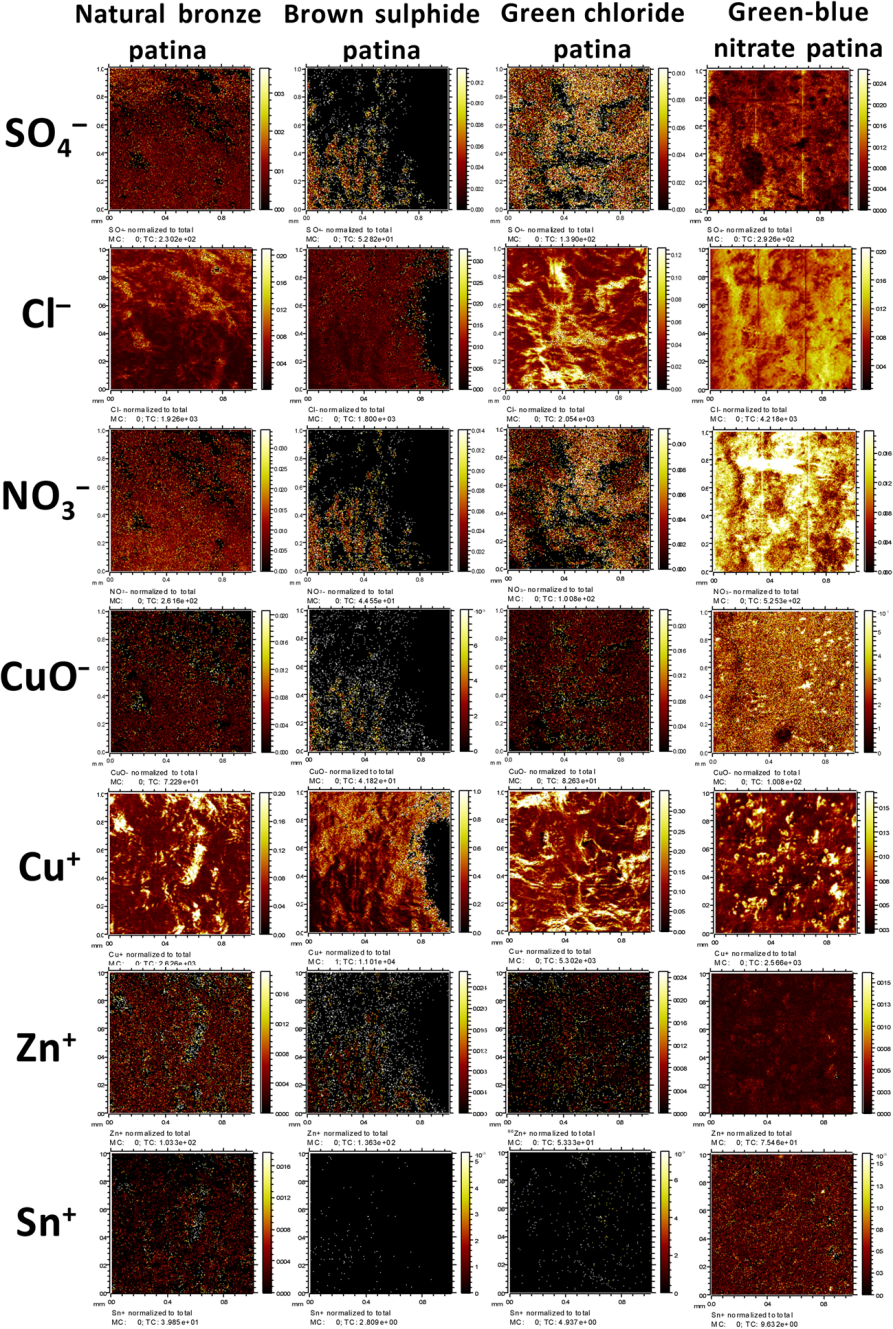
ToF–SIMS imaging in negative (the signals for SO_4_^−^, Cl^−^, NO_3_^−^, CuO^−^) and positive (the signals for Cu^+^, Zn^+^, and Sn^+^) polarities on an area of 1 mm by 1 mm

Figure 5 shows the distribution of different species on four different patina samples. The ToF–SIMS images for the SO_4_^−^, Cl^−^, NO_3_^−^, and CuO^−^ signals represent the 2D distribution of SO_4_^2−^, Cl^−^, NO_3_^−^, and Cu_2_O on the surface, respectively. On the other hand, Cu^+^, Zn^+^, and Sn^+^ originate from Cu-, Zn-, and Sn-containing species, respectively. The latter signals represent either metals or their oxides. It has to be pointed out that the detection limit of the ToF–SIMS technique is significantly lower than that for XPS, and consequently, the species that were not able to be analysed with XPS (results represented below) were detected by ToF–SIMS.

The spatial distribution of species that are present in urban rain (SO_4_^2−^, Cl^−^, NO_3_^−^) can be observed on all four patinas. High Cl^−^ intensity was found on green chloride patina and NO_3_^−^ on green–blue nitrate patina. On green–blue nitrate patina, SO_4_^2−^ and Cl^−^ were also distributed across the entire surface. The distribution of Cu^+^, Zn^+^, and Sn^+^ signals for natural brown patina most likely reflects the microstructure of bronze. The intensity of Zn^+^ and Sn^+^ signals is lower for the patinas that were formed artificially suggesting a lower concentration of these species on the outermost surface.

**Fig. 6:**
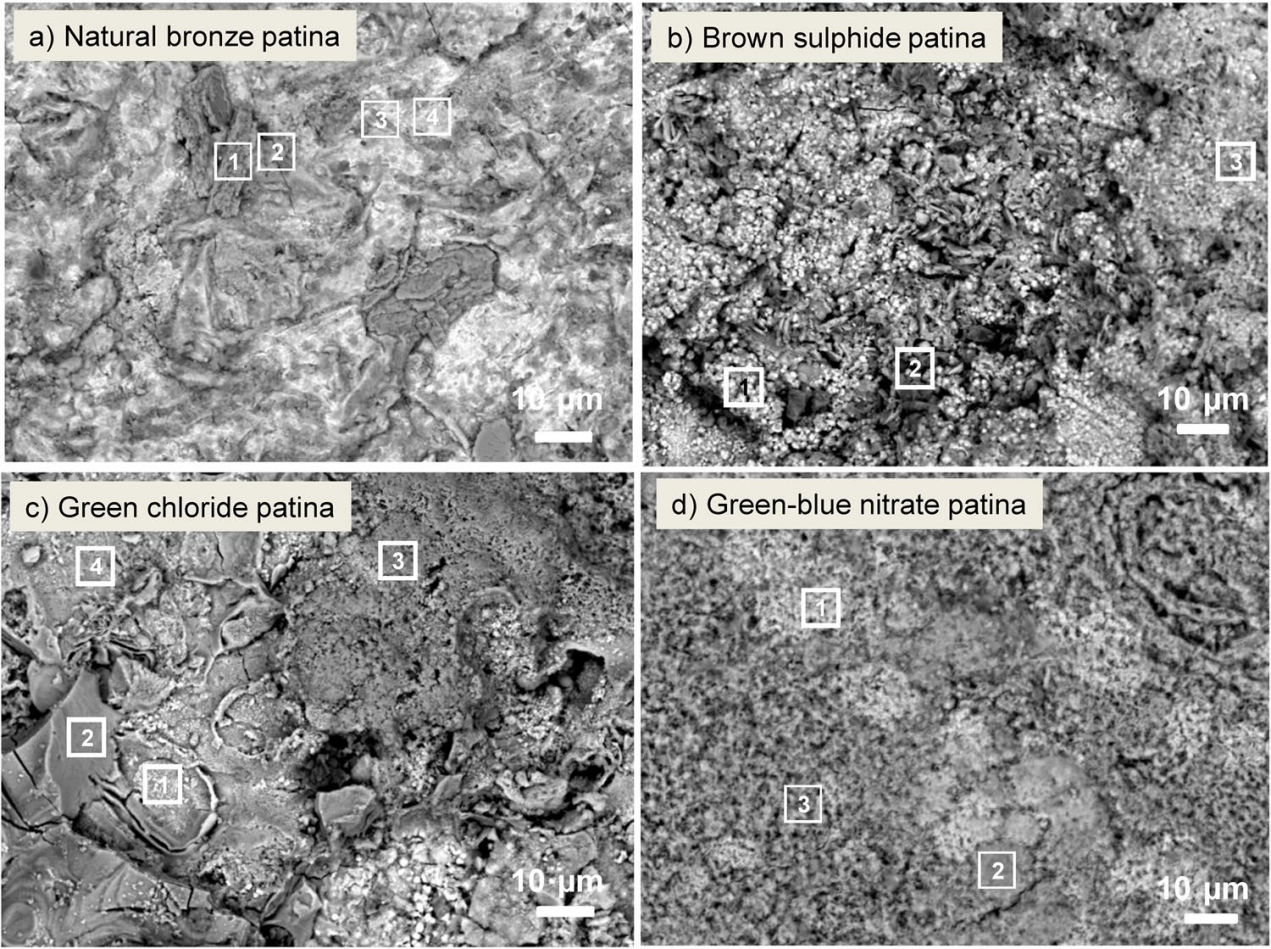
SEM images of the **a** natural bronze patina, **b** brown sulphide patina, **c** green chloride patina, and **d** green–blue nitrate patina

Figure 6 shows SEM images of the natural, brown sulphide, green chloride, and green–blue nitrate patinas after 9-year exposure to the urban environment. EDXS analyses were performed at the locations denoted by numbered squares on the SEM images. The EDXS elemental analysis (in at.%) is presented in Table 3.

**Table 3:** EDXS analysis at different locations on the patina surface after 9 years of urban environment exposure. The values represent an average value ± standard deviation. All values are given in weight%

Patina	Area	Cu	O	S	Cl	C	Sn	Si
Natural bronze patina	1	44.6 ± 1.2	39.3 ± 1.2	5.3 ± 0.4	0.6 ± 0.2	9.5 ± 1.7	nd	0.4 ± 0.2
	2	28.0 ± 0.3	48.1 ± 0.4	2.0 ± 0.2	nd	7.5 ± 0.5	nd	4.0 ± 1.8
	3	44.4 ± 0.4	35.6 ± 0.2	2.0 ± 0.1	1.1 ± 0.2	10.0 ± 0.1	3.1 ± 0.1	0.9 ± 0.3
	4	55.0 ± 5.5	22.0 ± 4.9	0.7 ± 0.5	0.8 ± 0.3	7.5 ± 1.5	8.0 ± 1.8	0.9 ± 0.2
Brown sulphide	1	58.4 ± 3.6	27.4 ± 0.7	1.8 ± 1.2	1.9 ± 0.6	9.7 ± 0.1	nd	nd
	2	44.4 ± 1.9	36.2 ± 0.6	6.4 ± 0.4	0.7 ± 0.2	8.0 ± 0.9	nd	nd
	3	56.7 ± 1.5	23.4 ± 1.3	5.6 ± 1.3	1.4 ± 0.1	10.8 ± 1.2	nd	nd
Green chloride	1	51.4 ± 0.8	28.3 ± 0.5	nd	9.3 ± 1.2	11.0 ± 1.6	nd	nd
	2	48.0 ± 4.2	29.1 ± 1.7	2.5 ± 1.1	11.7 ± 9.0	7.4 ± 0.1	0.6 ± 0.1	≥ 1
	3	49.1 ± 0.2	38.1 ± 0.1	0.2 ± 0.1	1.1 ± 0.1	11.4 ± 0.2	nd	nd
	4	51.8 ± 1.9	29.5 ± 2.1	2.4 ± 1.5	6.1 ± 1.6	8.8 ± 0.4	nd	0.7 ± 0.2
Green–blue nitrate	1	47.8 ± 0.8	25.1 ± 1.0	0.6 ± 0.1	13.7 ± 1.8	11.2 ± 1.1	nd	nd
	2	49.1 ± 0.9	42.8 ± 1.0	1.6 ± 0.4	1.2 ± 0.3	7.8 ± 0.7	nd	nd
	3	48.3 ± 5.8	40.3 ± 1.3	0.5 ± 1.0	1.6 ± 0.3	6.2 ± 1.0	nd	nd

*nd*, not detected (below the detection limit).

Plane-like crystals appear in the natural bronze patina, and their composition (area 1 in Table 3) and morphology suggest the presence of copper sulphate crystals, as has been reported previously for corrosion products in urban rain (Kosec et al. 2012). Furthermore, a similar composition was determined, although the Si content was higher, which could indicate the deposition of Si at the surface and the initiation of brochantite formation (defined below with Raman spectroscopy analysis) at points of surface irregularity (area 2, Fig. 6a). Areas 3 and 4 (Fig. 6a) differ with respect to the amount of Cu and Sn at the surface.

Three different regions were observed on the brown sulphide patina after 9 years of urban environment exposure. Fine, rounded agglomerates consist of Cu, O, S, Cl, and C. The morphology of the surface products suggests the presence of Cu_2_S (area 1, Fig. 6b), as has been shown previously (Masi et al. 2017). Area 2 in Fig. 6b is similar to area 1 in Fig. 6a, indicating the presence of copper sulphate hydroxy-minerals. In terms of composition and morphology, area 3 is similar to area 1.

In the green chloride patina, 4 different morphologies were observed: a burst (area 1), a smooth surface beside the burst (area 2), a flat surface (area 3), and a very rough area (area 4), occupying larger portions of the surface. Each area contained Cl, while a higher amount of S was determined in the smooth area (area 2)—although the amount was still lower than that found on the natural and brown sulphide patinas.

A fine-grained area containing Cl (area 1, Fig. 6d) was found in the green–blue nitrate patina, while areas 2 and 3 revealed the presence of Cl and S (Fig. 6d). The presence of Cl is the result of environmental exposure, while S is present due to the underlying sulphide patina applied as part of the sample preparation process. No S-rich areas were found, with around 6 at.% of S, indicating the absence of minerals containing sulphur. This is investigated further through Raman spectroscopy and XRD analyses presented below.

### XRPD measurements

The XRD pattern of the natural bronze patina after 9 years of urban environment exposure showed the presence of well-resolved peaks, indicating the underlying copper substrate (peaks at 2θ angles 42.9° and 49.9° 2θ), and Cu_2_O, with low intensity reflections visible at 36.4°, 42.9°, and 61.3° 2θ, as presented in the XRPD spectra in Fig. 6. Both Cu substrate and cuprite were also detected in all three of the artificially patinated bronze surfaces (the brown patina, green chloride patina, and green–blue nitrate patina). The presence of copper sulphide (Cu_2_S, chalcocite) was confirmed in the brown (sulphide) patina, with peaks identified at 37.2°, 45.8°, 48°, and 53.7° 2θ. These peaks had a significantly lower intensity than those for Cu metal (due to the substrate) and cuprite. Similar observations have been reported previously, with the low intensity of the peaks most likely being due to the poorer crystallinity of Cu_2_S (Ropret and Kosec 2012).

**Fig. 7:**
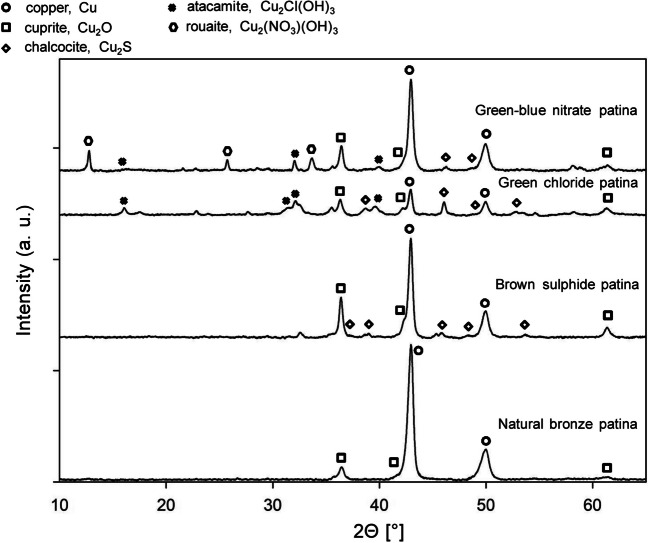
XRPD diffractograms of the various patinas developed over the course of 9-year urban environment exposure: (a) natural bronze patina, (b) brown sulphide patina, (c) green chloride patina, and (d) green–blue nitrate patina

The XRD pattern of the green chloride patina revealed the presence of atacamite, Cu_2_Cl(OH)_3_, with reflections at 16.1°, 17.6°, 31.5°, 32.2°, and 39.7° 2θ (ranked in descending order of intensity), which most probably relates to the presence of atacamite. These peaks are also present in the XRD spectra of the green–blue nitrate patina. The presence of atacamite in a green–blue nitrate patina has not been observed previously and needs to be explained further. In addition to atacamite, peaks indicating the presence of chalcocite are also visible (peaks at 38.7°, 46.0°, 49.9°, and 54.8°). The intensity of the peaks identifying atacamite and chalcocite are similar to those of the Cu substrate and Cu_2_O, probably related to the formation of a thick, homogeneous patina layer on the substrate. XRD patterns of the green–blue nitrate patina showed peaks defined at 12.7°, 25.6°, and 36.4° 2θ, can be ascribed to rouaite, Cu_2_(NO_3_)(OH)_3_. Atacamite peaks are also shown in the XRPD spectra of the green–blue nitrate patina, in addition to peaks for Cu substrate and Cu_2_O (Fig. 7).

### Raman spectroscopy analysis

**Fig. 8:**
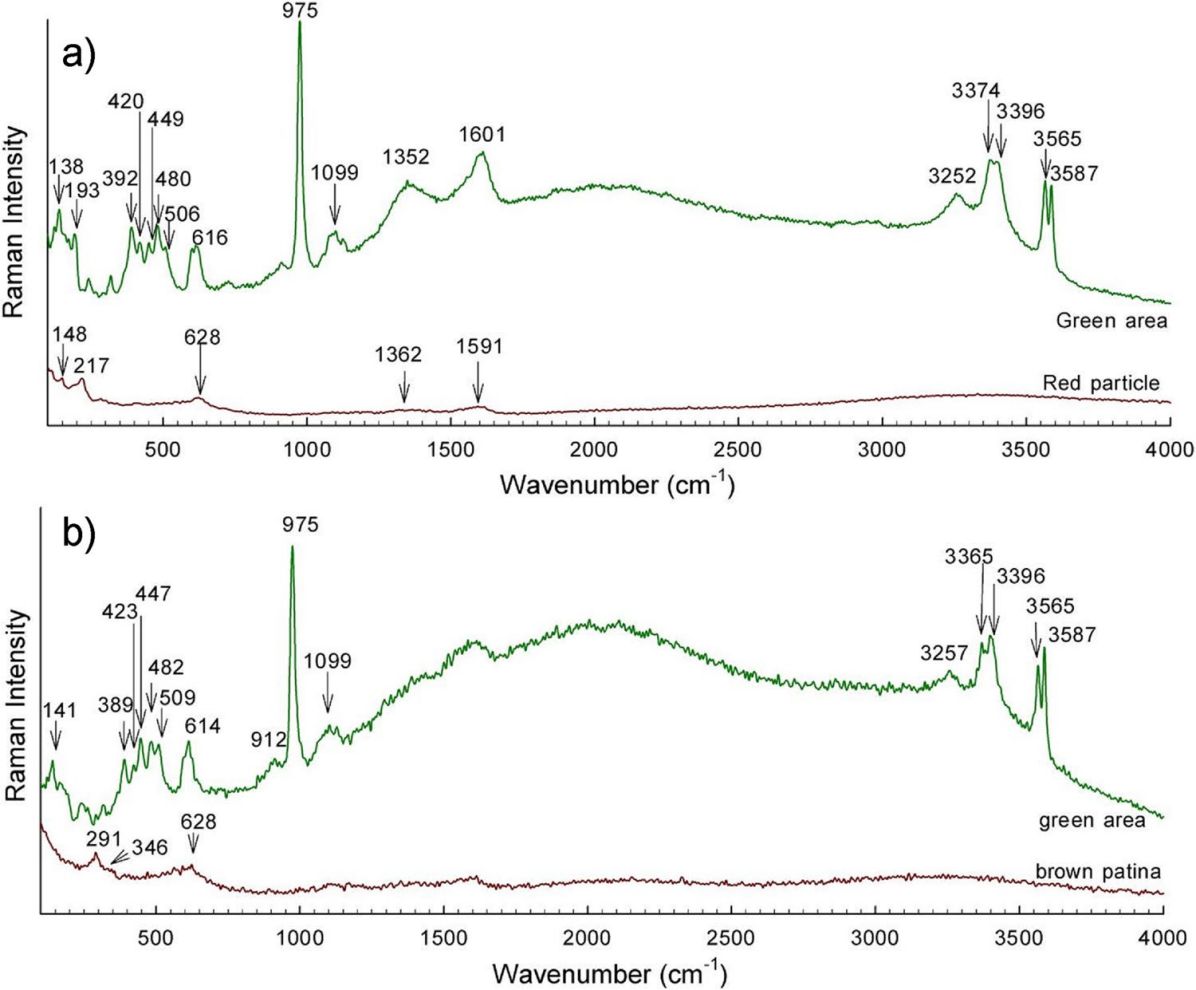
Raman spectra at different areas of **a** the natural bronze patina and **b** the brown sulphide patina

**Fig. 9:**
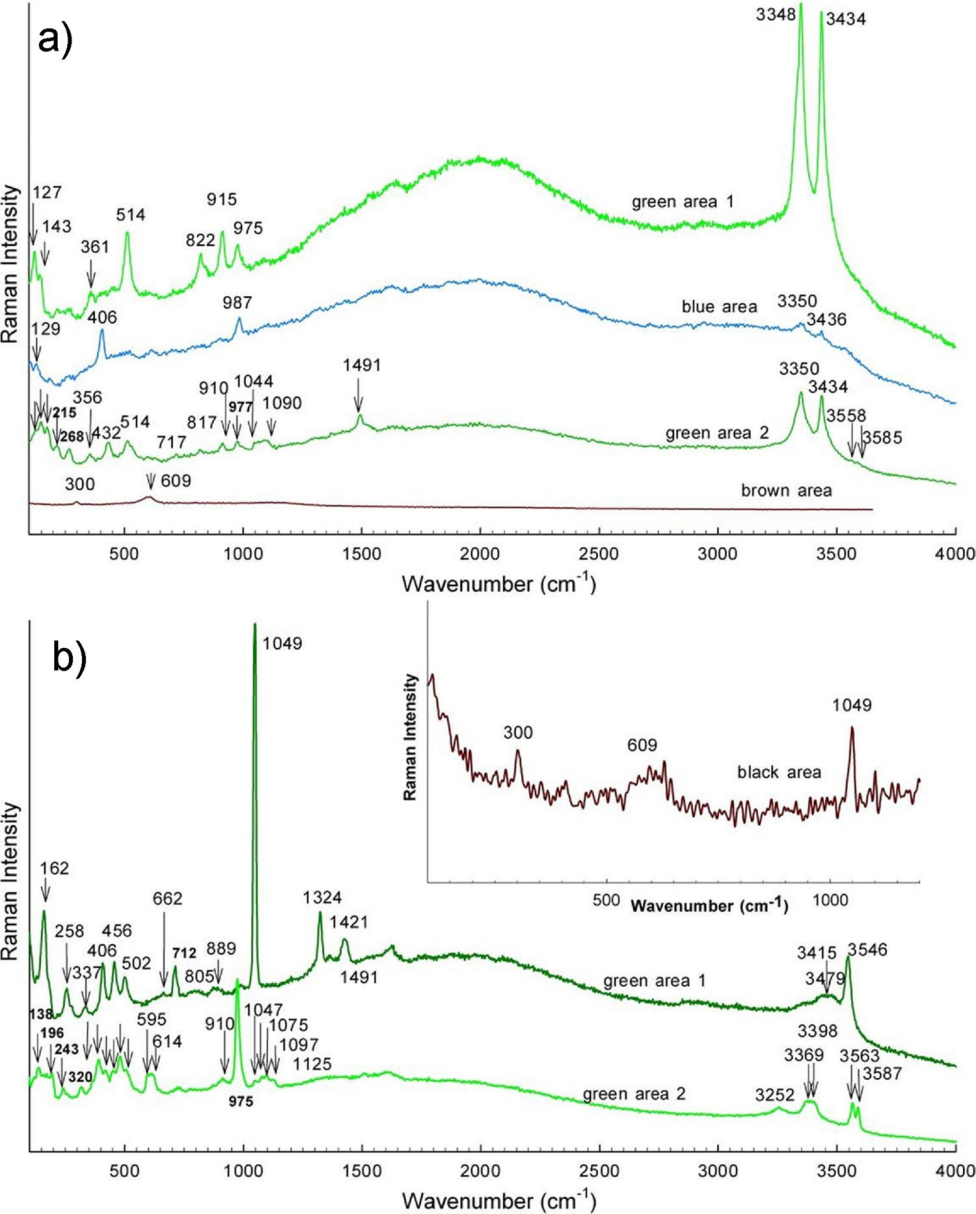
Raman spectra at different areas of **a** the green chloride patina and **b** the green–blue nitrate patina

Raman spectra were collected at the surface of the patinated bronze samples, and the results are gathered in Figs. 8 and 9. Raman bands are collected in Table S2 in Supplementary file. The surface of the natural bronze patina contains reddish-brown and green areas. The reddish-brown areas gave Raman modes at 1591, 1362, 628, 217, and 148 cm^−1^ (Fig. 8a, ‘red particle’). The first two modes belong to carbon (Bell et al. 1997) particles deposited at the surface over the 9 years the sample was exposed to the urban environment, while the modes at 628, 217, and 148 cm^−1^ indicate the presence of poorly crystallised Cu_2_O (McCann et al. 1999; Chan et al. 1999; Kudelski and Pettinger 2004; Debbichi et al. 2012; Ospitali et al. 2012; Špec et al. 2014). The green areas suggest the occurrence of brochantite (Hayez et al. 2005), with bands at 3587, 3565, 3396, 3373, 3252, 1119, 1099, 1080, 975, 616, 605, 507, 480, 449, 420, 392, 363, 241, 193, and 138 cm^−1^ (Fig. 8a, green area). Furthermore, the two broader bands centred around 1600 and 1350 cm^−1^ indicate the presence of carbon particles deposited on the green areas.

Dark brown and green areas can be seen on the surface of the brown sulphide patina when observed at higher magnifications. The Raman spectrum of the dark brown areas shows broad bands centred at 628, 346, and 291 cm^−1^ (Fig. 8b, brown sulphide patina). These modes are in agreement with bands of tenorite (CuO) (Debbichi et al. 2012), but the broad nature of the bands, especially the A_g_ mode at 291 cm^−1^ and the B_g_ mode at 628 cm^−1^, suggests they overlap with the bands of cuprite and chalcocite (McCann et al. 1999) that are usually found at 610, 325, and 280 cm^−1^. Moreover, chalcocite is usually the main constituent of a brown sulphide patina on bronze (Ropret and Kosec 2012). Similar to the case of the bare bronze, the green areas exhibit Raman modes significant for brochantite (Fig. 8b, green area).

Raman bands are present in the green chloride patina corresponding to both the initially formed patina and to degradation products. In Fig. 9a, ‘green area 1’ shows Raman modes at 3434, 3348, 975, 915, 822, 514, 360, 143, and 122 cm^−1^ belonging to atacamite Cu_2_Cl(OH)_3_, which is usually found on bronze samples treated to form an artificial chloride patination (Hayez et al. 2005). In addition to atacamite, other green areas (Fig. 9a, ‘green area 2’) show additional bands at 1491, 1090, 1044, 717, 530, 432, 356, 267, 215, 176, and 148 cm^−1^ (denoted by arrows) that belong to malachite, Cu_2_(CO_3_)(OH)_2_ (Bell et al. 1997; Špec et al. 2014). Two very weak modes at 3585 and 3558 cm^−1^ also indicate the possible presence of brochantite (denoted by arrows). The brownish-black surface showed two Raman modes at 609 and 300 cm^−1^, which indicates the presence of tenorite (Debbichi et al. 2012).

Tenorite can also be found on the black surfaces of the samples of green–blue nitrate patina at the surface (Fig. 9b, ‘black area’). The additional band at 1049 cm^−1^ belongs to the symmetric stretching vibration of NO_3_^−^, which is the strongest band of rouaite (Frost et al. 2004). The presence of rouaite in the green areas is further confirmed by Raman modes at 3546, 3479, 3415, 1422, 1324, 1049, 889, 805, 712, 662, 502, 456, 406, 337, 279, 258, and 162 cm^−1^ (see Fig. 9b, ‘green area 1’). Most probably, only rouaite is present, while other green areas show the formation of brochantite (Fig. 9b, ‘green area 2’) (Hayez et al. 2005).

### XPS measurements

**Fig. 10:**
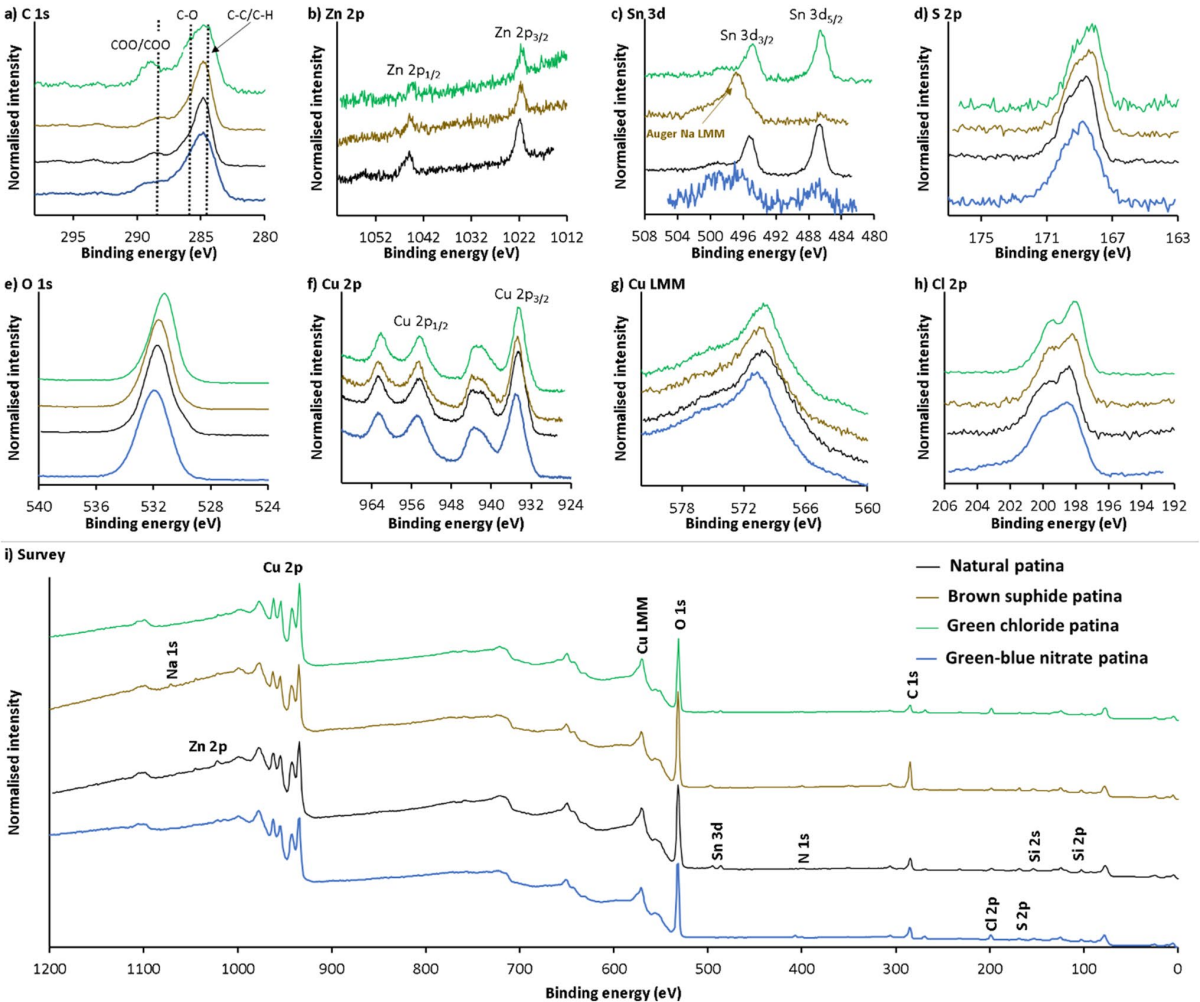
HR XPS spectra for **a** C 1 s, **b** Zn 2p, **c** Sn 3d, **d** S 2p, **e** O 1 s, **f** Cu 2p, **g** XPS-induced Auger Cu LMM, and **h** Cl 2p, and **i** survey spectra of the aged natural, brown sulphide, and green chloride patinas

Figure 10 shows HR XPS and survey spectra for the natural bronze, brown sulphide, green chloride, and blue-green nitrate patinas prior to sputtering. The surfaces comprise C-containing species composed of C–C/C-H, C-O, COO/COOH, and carbonates (Fig. 10a), which most likely originate from the soot present in the surrounding environment. The contribution to these signals may also come from the adventitious carbonaceous species adsorbed on the surface. Zn-, Sn-, S-, O-, Cu-, and Cl-containing species were present on all the sample surfaces, apart from missing Zn for green–blue nitrate patina. The amount of Zn on these surfaces was low (less than 1.1 at.% in all cases, as shown in Fig. 10b), resulting in higher spectral noise in Fig. 10b. The position of the Sn 3d_5/2_ peaks at 486.5–485.7 (Fig. 10c) indicates the formation of SnO_2_ and/or SnO (Chang et al. 2019). However, the peaks for Sn 3d_5/2_ and Sn 3d_3/2_ were also barely visible for green–blue nitrate patina. In the brown sulphide patina, the Sn 3d_5/2_ peak is also barely visible, as the amount of Sn-containing species on the surface was 0.2 at.%. Moreover, in the same sample, the Sn 3d_3/2_ peak overlaps with the XPS-induced Auger Na LMM peak (the presence of Na is also confirmed in the survey spectra by the presence of the Na 1 s peak). The depth profile presented below for that particular sample was performed at a different location on the sample, where the Auger Na LMM signal did not develop. The position of the peaks in the S 2p spectra in Fig. 10d indicates that the sulphur is oxidised, probably in the form of sulphates (Laibinis et al. 1991; Hosseini et al. 2005; Fonder et al. 2008; Finšgar 2013; Finšgar and Merl 2014). The O signals seen in Fig. 10e could have originated from various species, i.e. metallic oxides/hydroxides, sulphates (as described above), and O from the oxidised carbonaceous species (malachite) in the chloride patina, as determined in the C 1 s spectra (see Fig. 9a). The presence of shake-up satellites in the Cu 2p spectra (Fig. 10f) and the shape of the XPS-induced Auger Cu LMM spectra suggest that the uppermost position of all the samples contained Cu(II) species (Finšgar 2018). All the peaks in the Cl 2p spectra were positioned at 198.6 eV (the main peak), as shown in Fig. 10h, confirming the presence of metal chlorides on the surfaces (Moulder et al. 1995).

## Discussion

The process of evolution of natural and artificially formed patinas is a result of three different factors:

i) Initial patinas’ properties and the patina thickness
ii) The composition of urban rain
iii) The competitive reactions between corrosive species in the environment, artificial patina, and the hydrophobic nature of the surfaces

### Initial patinas’ properties and the patina thickness

We have shown by electrochemical measurements that after 9 years of exposure to the urban environment, natural patina on bronze had more protective properties than brown sulphide, green chloride, and green–blue nitrate patina as observed from polarisation resistance values measured in concentrated urban rain (Fig. 2, Table 2). The brown sulphide patina had the lowest *R*_p_ value, assumingly due to the process of forming a secondary patina in the form of cupric sulphates. Namely, our previous results showed that lower *R*_p_ values were measured on bronze and patinas immediately after immersion (Kosec et al. 2010b, a; Fabjan et al. 2011). In general, the *R*_p_ values of natural patina increased, which means it is less susceptible to corrosion.

### Composition of urban rain

The composition of the acid rain as the prevailing environmental condition was taken into consideration. The average composition of rain in the Ljubljana region over 9-year period was 0.686 mg/L SO_4_^2−^, 0.287 mg/L Cl^−^, and 0.943 mg/L NO_3_^−^. The amount of sulphates in this urban rain is lower than nitrates while, most importantly, also chlorides are present in the studied urban environment, which affected the patinas’ evolution. When outdoor exposure is compared to other accelerated ageing, the following findings can be enlightened. In our previous study, the urban environment was resembled through ageing in the climatic chamber consisting of 3 weeks of SO_2_ exposure, 9 weeks of humidity exposure, and 48-h exposure to chlorides in salt spray (Ropret and Kosec 2012). Paratacamite, clinoatacamite, and atacamite developed on all of the samples, while brochantite was found only on brown sulphide patina. When ageing was executed by exposure to simulated urban rain, consisting of Na_2_SO_4_, NaHCO_3_, and NaNO_3_, brochantite and langite, Cu_4_(SO_4_)(OH)_6_·2H_2_O, were present on all the patinas due to the absence of chlorides in this environment (Kosec et al. 2012). The results of the present study show that neither previously used method, such as exposure to a climatic chamber, nor immersion in urban rain can be compared to exposure in an urban environment and that the results of 9-year exposure to an urban environment are very valuable. Namely, SO_4_^2−^, Cl^−^, and NO_3_^−^ species all affected the change of natural bronze patina and artificially formed patinas throughout exposure time.

### Competitive reactions between corrosive species in the environment, artificial patina, and the hydrophobic nature of the surfaces

Brochantite Cu_4_SO_4_(OH)_6_ is usually a constituent of patinas developed following exposure to an environment polluted with SO_2_. Similarly, antlerite and posnjakite can be found, depending on the conditions of the site and the length of exposure (Hayez et al. 2004; Chiavari et al. 2007; Robbiola et al. 2008; Bernardi et al. 2009; Kosec et al. 2021).

When the concentration of cupric and sulphate ions in the electrolyte are high enough to form brochantite, it starts to precipitate on the Cu_2_O (Aastrup et al. 2000; Krätschmer et al. 2002; Clarelli et al. 2014). A similar process is evidently established on the cuprous sulphide, green chloride, and green–blue nitrate patinas, since brochantite was also found on each of these patinas (Zittlau et al. 2013).

In the case of green–blue nitrate patina, rouaite, brochantite, and atacamite were found. The lowest standard free energy (ΔG_f_^⦵^_298 K_) had brochantite at − 2158.1 ± 7.0 kJ/mol (Woods and Garrels 1986; Bureš et al. 2020), rouaite Cu(NO_3_)_2_·3H_2_O had − 1217 kJ/mol (Varma et al. 2016), and atacamite had − 1340 kJ/mol (Varma et al. 2016). Thus, thick and compact appearance and minimal change in 9-year exposure of the green–blue nitrate patina is not due to standard free energy of formation but could be explained through the high thickness of the layer (30 µm thick; Fig. 3).

**Fig. 11:**
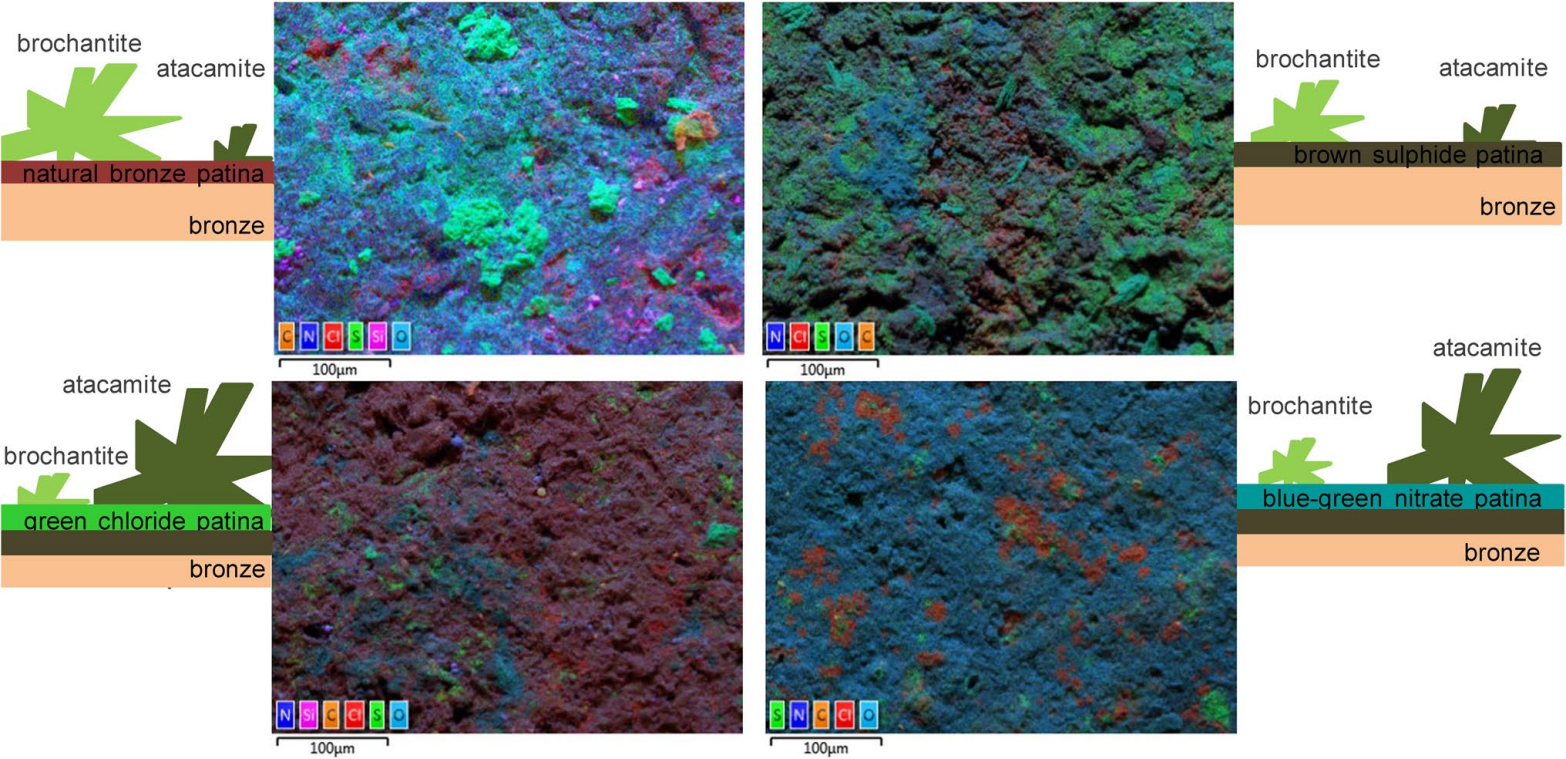
Schematic presentation of combination of EDXS maps and deduced estimation of brochantite and atacamite occurrence and abundance on the bare bronze, brown sulphide, green chloride, and green–blue nitrate patina after 9-year exposure

Figure 11 presents EDXS maps showing elemental distribution of C, O, S, and Cl together with a simplified sketch of the abundance of atacamite and brochantite; the larger the plotted crystal, the higher the estimated abundance over exposed surface. The presence of S with typical morphological clustering in the form of plate like crystals points at the presence of brochantite while presence of Cl shows possible presence of atacamite. Atacamite was not identified by XRD on natural bronze patina and brown patina, nor was it determined by Raman spectroscopy. Cl^−^ as atacamite constituent was, however, analysed by XPS and ToF–SIMS as these two techniques are more surface sensitive techniques. Figure 11 shows that atacamite was identified in higher abundance on the nitrate patina when compared to presence of brochantite (higher intensity of Cl). This could be explained by the fact that the contact angle on nitrate patina was the lowest at 51.5 ± 3.2°. The urban rain, which in the case of Ljubljana rain, contained chloride ions had thus longer contact time and higher contact surface, thus forming more atacamite than on other patinas such as natural bronze patina and brown sulphide patina.

The most significant finding of the study is that after 9 years of exposure to urban environment, the highest amount of brochantite was found on the patina that had naturally developed on the bare bronze. Brochantite was less abundant on the sulphide brown and green chloride patinas and even less so on the nitrate patina.

## Conclusions

Bare bronze and artificially produced brown sulphide, green chloride, and green–blue nitrate patinas were exposed to an urban environment containing sulphate, nitrate, and chloride anions for 9 years. The patinas that developed over 9 years of urban environment exposure were thoroughly investigated using a multi-analytical approach. The following conclusions can be drawn:

1. After 9 years of urban environment exposure, the 5–10 µm thick natural patina developed on bronze. The visual assessment showed that the brown sulphide patina did not change much over 9 years of exposure. The surface of green chloride patina changed the most with very heterogeneous appearance due to poorly adhered artificial patina. The green–blue nitrate patina remained thick and compact.
2. Stratigraphic analysis, X-ray mapping, and morphological characterisation determined by SEM and ToF–SIMS imaging showed that the patinas are very heterogeneous, i.e. it was possible to observe the impact of both the preparation method and the environmental exposure on the outer layer of patinas.
3. Brochantite was formed on all the patinas investigated, as confirmed by Raman spectroscopy. The presence of atacamite was identified on the green–blue nitrate patinas as a result of the hydrophilic nature of the patina and the presence of chlorides in the urban environment and on the green chloride patina due to its chemical origin.
4. When patina is poorly crystallised, both XRPD and Raman spectroscopy analysis could not give sufficient proof of the presence of the patina constituents. Through XPS and ToF–SIMS investigation, it was possible to identify the main ion species in the uppermost patina layer; identifying the constituents of the patina is very important for the design of future protection systems.

The authors acknowledge the inherent limitations of the present study, as it exclusively focused on a singular region and examined bronze surfaces that were consistently oriented in the same direction. Subsequent investigations may benefit from incorporating colourimetric measurements, contact angle assessments, and analyses conducted on reduced-size specimens. This strategic approach aims to delineate the temporal progression of corrosion product development, thereby surpassing the confines of a conclusive final analysis.

### Supplementary Information

Below is the link to the electronic supplementary material.

Supplementary file1 (JPG 571 KB)

Supplementary file2 (DOCX 14.6 KB)

## Data Availability

The data that support the findings of this study are available from the corresponding authors upon reasonable request.
