# Supplementary figures and images for: The impact of urban rain on the changes of bare and artificially patinated bronze during 9-year exposure
Source: Environ Sci Pollut Res Int. 2024 Apr 19;31(22):31925–41. doi: 10.1007/s11356-024-33369-9 (PMC11133102; doi:10.1007/s11356-024-33369-9)

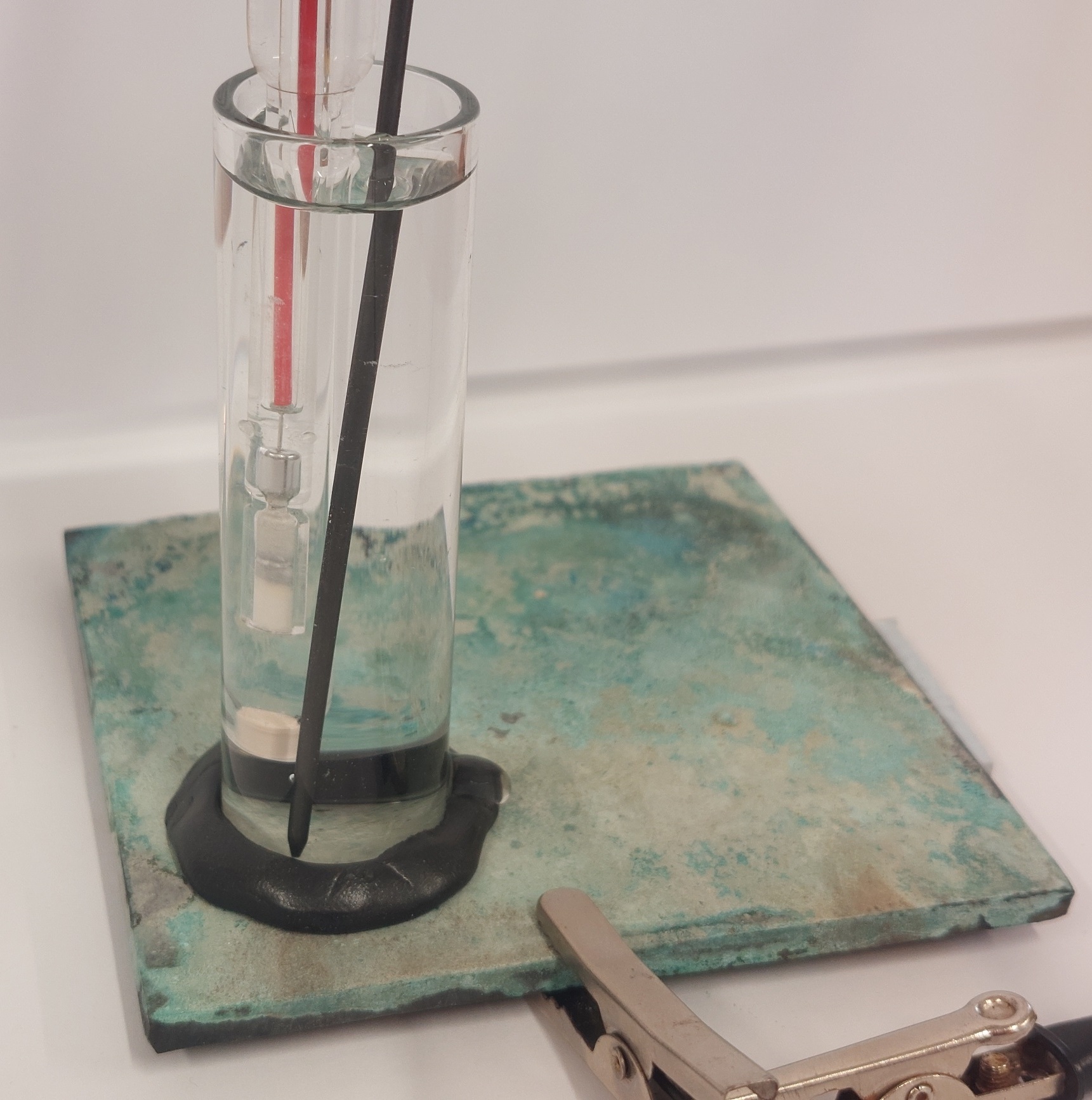

Supplement: Supplementary file 1 — Supplementary file1 (JPG 571 KB) [file 11356_2024_33369_MOESM1_ESM.jpg]
