# Supplementary material for: The impact of urban rain on the changes of bare and artificially patinated bronze during 9-year exposure
Source: Environ Sci Pollut Res Int. 2024 Apr 19;31(22):31925–41. doi: 10.1007/s11356-024-33369-9 (PMC11133102; doi:10.1007/s11356-024-33369-9)
Supplement: Supplementary file 2 — Supplementary file2 (DOCX 14.6 KB) [file 11356_2024_33369_MOESM2_ESM.docx]

Table S2. Raman bands (cm^–1^), identified on natural bronze patina, brown sulphide patina, green chloride patina and blue-green nitrate patina. Leters next to a number denote strength of the band: vw (very weak), w(weak), s (strong) and vs (very strong).

| Natural bronze patina | | Brown sulphide patine | | Green chloride patina | | | Blue-green nitrate patina- | | |
| --- | --- | --- | --- | --- | --- | --- | --- | --- | --- |
| Cu_2_O | brochantite | chalcocite | brochantite | atacamite | malachite | tenorite | tenorite | rouaite | brochantite |
|  |  |  |  | 122 |  |  |  |  |  |
| 148 | 138 |  | 141 | 143 | 148 |  |  |  | 138 |
| 217 | 193 |  | 193 |  | 176 |  |  | 162 (s) | 193 |
|  | 241 | 291 | 241 |  | 215 | 300(s) | 300 (w) | 258 | 241 |
|  | 363 | 346 | 363 |  | 267 |  |  | 279 | 363 |
|  | 392 (s) |  | 389 (s) |  |  |  |  |  | 392 |
|  | 420 |  | 420 | 360 | 356 |  |  | 337 | 420 |
|  | 449 |  | 447 (s) |  | 432 |  |  | 406 | 449 |
|  | 480 |  | 480 |  |  |  |  | 456 | 480 |
|  | 507 |  | 507 | 514 | 530 |  |  | 502 | 507 |
| 628 | 605 |  | 605 |  |  | 609 (s) | 609 (w) | 662 | 595 (s) |
|  | 616 (s) | 628 | 614 (s) |  | 717 |  |  | 712 | 614 (s) |
|  |  |  |  | 822 |  |  |  | 805 |  |
|  |  |  |  | 915 |  |  |  | 889 |  |
|  | 975 (vs) |  | 975 (vs) | 975 |  |  |  |  | 975 (s) |
|  | 1080 |  | 1080 |  | 1044 |  |  | 1049 (vs) | 1080 |
|  | 1099 |  | 1099 |  | 1090 |  |  |  | 1099 |
|  | 1119 |  | 1119 |  |  |  |  | 1324 (s) | 1119 |
|  |  |  |  |  | 1491 |  |  | 1421 (s) |  |
|  |  |  |  |  |  |  |  |  |  |
|  | 3252 |  | 3252 | 3348 (vs) |  |  |  | 3415 | 3252 |
|  | 3373 (s) |  | 3373 |  |  |  |  |  | 3373 (s) |
|  | 3396 (s) |  | 3396 (s) | 3434 (vs) |  |  |  | 3479 | 3396 (s) |
|  | 3565 (s) |  | 3565 |  |  |  |  | 3546 (s) | 3565 (s) |
|  | 3587 (s) |  | 3587 (s) |  |  |  |  |  | 3587 (s) |
